# ATGL sensitizes hepatocellular carcinoma cells to genotoxic drugs by modulating p53 acetylation/phosphorylation status

**DOI:** 10.1038/s41420-026-03048-4

**Published:** 2026-03-20

**Authors:** Serena Castelli, Angela De Cristofaro, Enrico Desideri, Emanuele Salvi, Fabio Ciccarone, Maria Rosa Ciriolo

**Affiliations:** 1 Department for the Promotion of Human Science and Quality of Life, San Raffaele Open University, Via di Val Cannuta, 247, 00166 Rome, Italy; 2 IRCCS San Raffaele Roma, 00166 Rome, Italy; 3 Department of Biology, University of Rome Tor Vergata, 00133 Rome, Italy; 4 Department of Life Sciences, Health and Health Professions, Link Campus University, Via del Casale di San Pio V, 44, 00165 Rome, Italy

**Keywords:** Cancer metabolism, Biochemistry

## Abstract

Hepatocellular carcinoma (HCC) accounts for approximately 90% of liver cancer cases. Few therapeutic options are available for HCC patients due to intrinsic drug resistance or the low efficacy of conventional chemotherapeutic drugs, including genotoxic agents. We previously demonstrated that adipose triglyceride lipase (ATGL) is downregulated in HCC and shows anti-neoplastic activity by affecting sensitivity to different therapeutic approaches. On the basis of this evidence, we assessed the contribution of ATGL activity to the modulation of the DNA damage response induced by genotoxic drugs. We modulated ATGL expression via overexpression and silencing in the presence of etoposide and doxorubicin, which are genotoxic drugs. The catalytic activity of ATGL was abrogated by a selective inhibitor (ATGListatin) or the overexpression of the ATGL catalytic mutant. To assess the DNA damage response, we evaluated the phosphorylation of H2AX histones and the post-translational modifications of p53. The sensitivity to genotoxic drugs was assessed by analyzing cell viability and molecular markers associated with cell cycle arrest and cell death. Our results demonstrate that ATGL enhances DNA damage in HCC cells in response to genotoxic stimuli. The underlying molecular mechanism involves ATGL-mediated activation of PPARα/p300 signaling. As a result, we observed an imbalance in p53 acetylation/phosphorylation status that restrains cell cycle arrest and DNA damage repair while promoting apoptotic cell death. In line with the in vitro findings, bioinformatic analyses revealed a strong correlation between ATGL and the PPARα/p300 axis and further demonstrated an enrichment of gene sets associated with cell cycle regulation and DNA damage response in ATGL-high HCC. In conclusion, ATGL levels can be used as a predictive marker of HCC sensitivity to genotoxic insults. The activation of this lipase, or downstream molecular signaling, may thus be exploited to increase the efficacy of chemotherapeutic treatments in HCC.

## Background

Genotoxic treatments, such as radiation and chemotherapy, are commonly employed as primary or adjuvant therapies in cancer treatment. Unlike normal cells, which respond to genotoxic insults by activating a G1 phase cell cycle checkpoint, cancer cells exhibit dysfunctional cell cycle regulation and are more sensitive to DNA damage [1]. Genotoxic drugs exert their effects through various mechanisms and can induce multiple types of DNA damage. One widely used mechanism involves the inhibition of topoisomerase enzymes, which are essential for relaxing supercoiled DNA to facilitate transcription and replication. Inhibitors of these enzymes trap topoisomerases in intermediate complexes, leading to DNA double-strand breaks in the case of topoisomerase II inhibitors (such as etoposide and doxorubicin) and single-strand breaks for topoisomerase I inhibitors [2]. The resulting DNA damage response is orchestrated by the transcription factor p53, which promotes cell cycle arrest to facilitate DNA repair or the activation of cell death pathways according to the extent of genotoxic injury [3].

We previously demonstrated the involvement of the rate-limiting enzyme of lipolysis, adipose triglyceride lipase (ATGL), and fatty acid oxidation in the proliferation of hepatocellular carcinoma (HCC) cells [4, 5]. ATGL activity has both metabolic and signaling-related consequences within the cell [6]. Metabolically, ATGL leads to a reduction in the size and number of lipid droplets, consequently enhancing mitochondrial β-oxidation. From a signaling perspective, ATGL-mediated lipolysis results in the release of free fatty acids (FAs), which can bind and activate downstream nuclear receptors, including members of the peroxisome proliferator-activated receptor (PPAR) family [7]. In this context, ATGL activity has been specifically linked to the activation of the transcription factor PPARα in the liver [8], and our previous findings support a positive correlation between ATGL expression and PPARα activity in HCC [4]. The nature of the ligand that activates PPARα plays a crucial role in modulating its transcriptional activity, ultimately shaping the specificity of the downstream cellular response. In HCC, PPARα regulates several key pathways, including fatty acid oxidative metabolism, energy expenditure, and the control of cell proliferation and apoptosis [9].

We also observed an association between ATGL lipolytic activity and sensitivity to different therapeutic approaches in HCC. These findings are particularly relevant given that ATGL expression is lower in HCC tissues than in normal liver tissues, highlighting ATGL as a potential therapeutic target and prognostic marker. Specifically, ATGL overexpression restrains the efficacy of glycolytic inhibitors and favors the cytotoxicity of genotoxic compounds. As the effect of glycolytic inhibitors was shown to be dependent on ATGL-mediated enhancement of oxidative metabolism, which attenuates the Warburg effect [4], in the present study, we aimed to explore the role of ATGL activity in regulating the cell response to DNA-damaging agents, such as etoposide and doxorubicin, with a particular focus on the role of ATGL lipolytic activity in signaling.

## Results

### ATGL overexpression enhances genotoxic drug-induced DNA damage

**Fig. 1:**
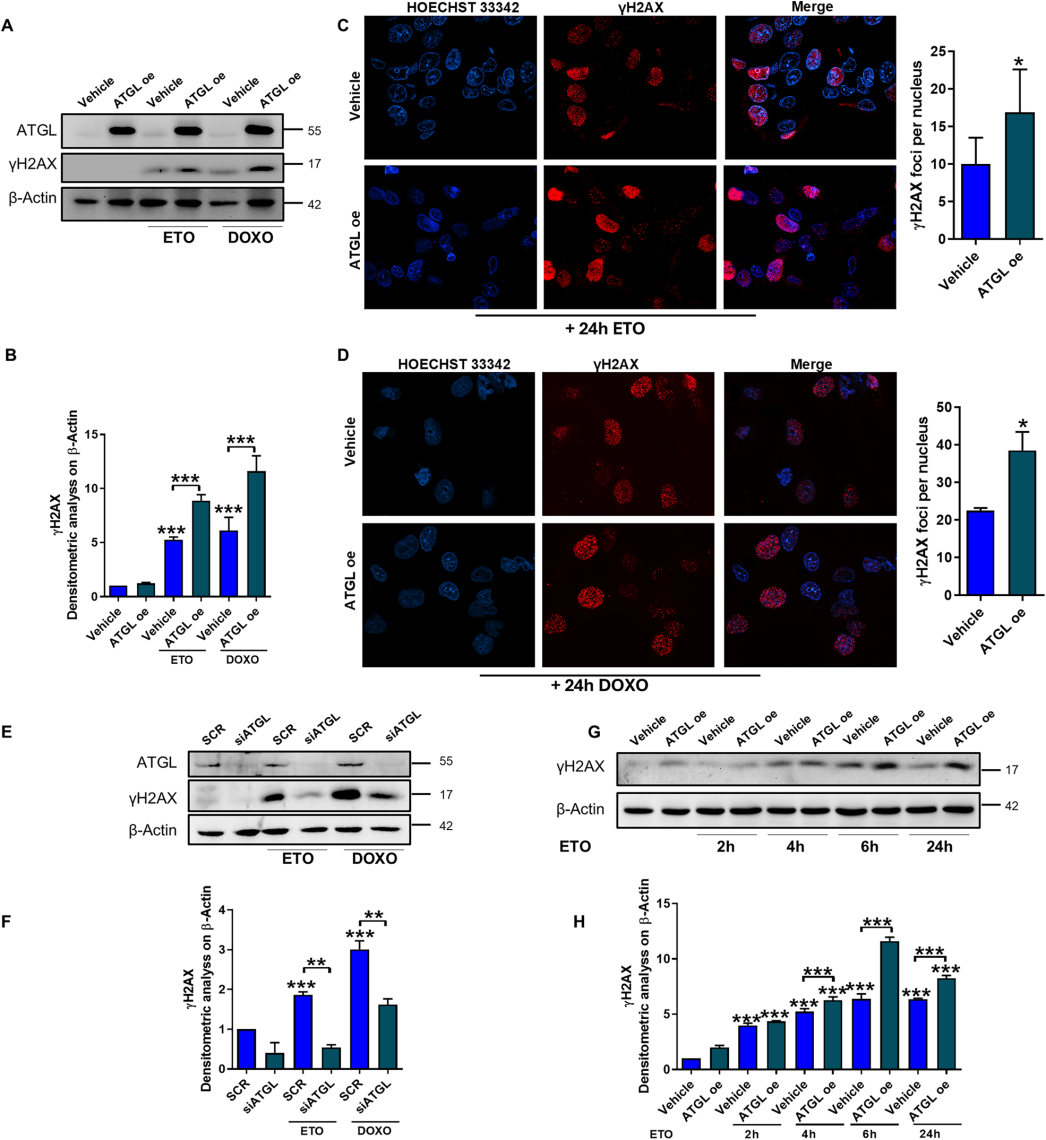
ATGL overexpression enhances genotoxic drug-induced DNA damage. HepG2 cells were transfected with empty vector (Vehicle) or ATGL-overexpressing construct (ATGL-OE) and, after 24 h, treated with 50 µM etoposide (ETO) or 2 µM doxorubicin (DOXO) for 24 h. **A**, **B** Western blot analysis of γH2AX levels was performed. Representative immunofluorescence images and quantification of γH2AX-foci per nucleus in HepG2 cells treated with etoposide (**C**) or doxorubicin (**D**). HepG2 cells were silenced for ATGL (siATGL; SCR Scramble) and, after 24 h, treated with 50 µM etoposide or 2 µM doxorubicin for 24 h. **E**, **F** Western blot analysis of γH2AX levels was performed. **G**, **H** HepG2 cells were transfected with Vehicle or ATGL-OE construct and treated with 50 µM etoposide for 2, 4, 6 or 24 h. Western blot analysis of γH2AX levels was performed. The images are representative of three independent experiments that yielded similar results. β-Actin and ATGL were used as loading and transfection controls, respectively. The data are presented as the means ± SDs from three independent experiments. Statistical significance was determined by one-way ANOVA with Tukey’s post hoc test; *p < 0.05, **p < 0.01, ***p < 0.001 vs CTRL or as indicated by brackets.

Our previous studies on the effects of ATGL in HCC revealed different susceptibilities to common therapeutic drugs, with ATGL-overexpressing cells being more resistant to glycolysis inhibitors than to genotoxic compounds. However, the impact of ATGL levels on the DNA damage response has not been explored in depth. We investigated the extent of DNA damage upon treatment with etoposide or doxorubicin (Sigma‒Aldrich, St. Louis, MO, USA) in HepG2 cells following ATGL modulation. We demonstrated that both compounds induced increased accumulation of phosphorylated histone H2AX (γH2AX) in ATGL-overexpressing HepG2 cells, as revealed by western blotting (Fig. 1A, B) and immunofluorescence (Fig. 1C, D), after 24 h of treatment. This relationship between ATGL expression and genotoxic DNA damage was further confirmed by the reduction in γH2AX levels following treatment with etoposide and doxorubicin in ATGL-silenced cells (Fig. 1E, F). Since γH2AX is an early event following genotoxic damage, a time-course experiment was performed to understand when ATGL modulates the kinetics of H2AX phosphorylation. We observed that the increased levels of DNA damage in ATGL-overexpressing cells were highly significant after 6 h of etoposide treatment (Fig. 1G, H), which was then used as the main time point in subsequent experiments.

### ATGL enzymatic activity is responsible for the increased DNA damage induced by etoposide

**Fig. 2:**
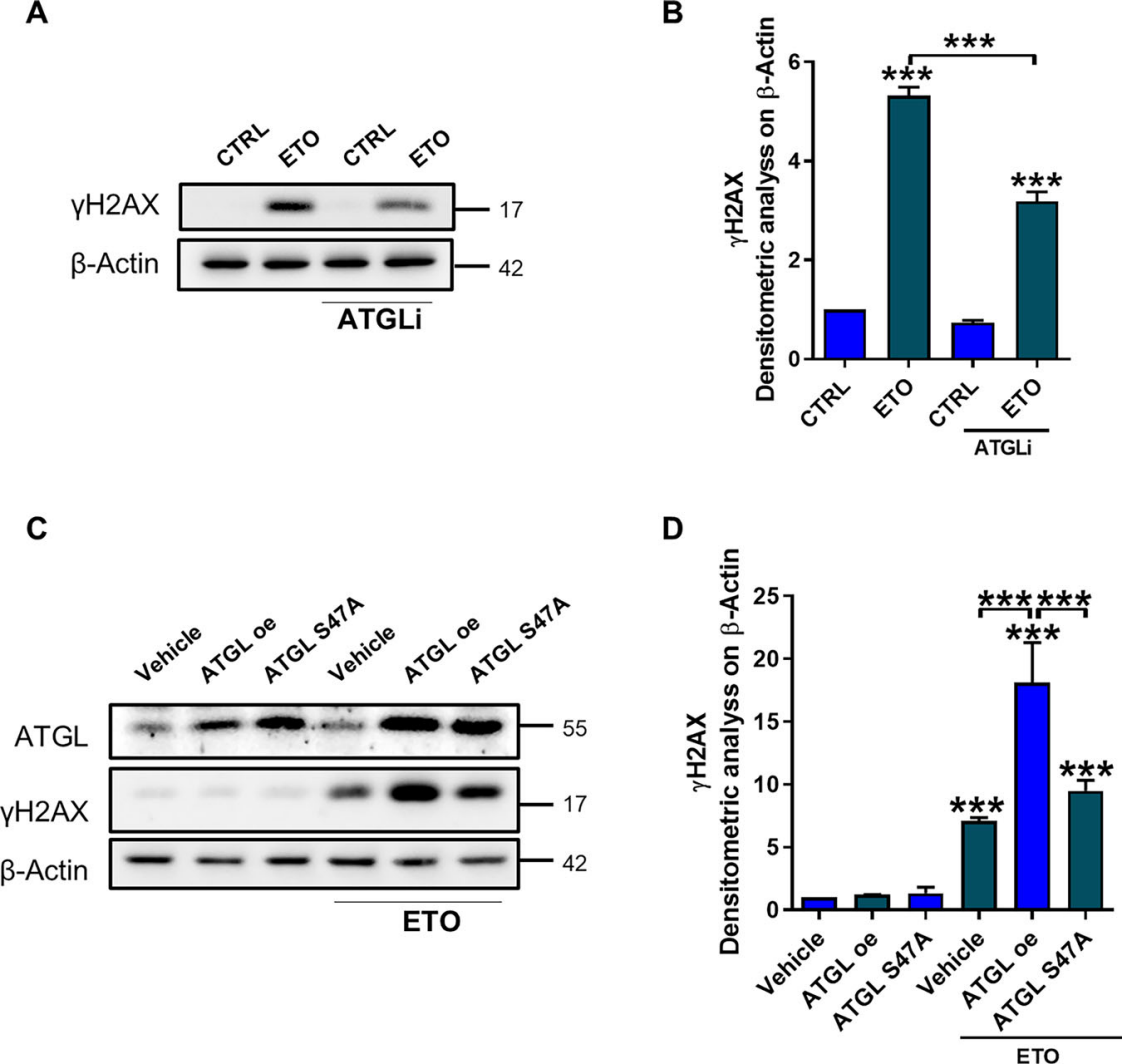
ATGL enzymatic activity is responsible for the increased DNA damage induced by etoposide. HepG2 cells were treated with 50 µM etoposide for 6 h with or without 25 µM ATGListatin (ATGLi) for 24 h. **A**, **B** Western blot analysis of γH2AX levels was performed. HepG2 cells were transfected with empty vector (Vehicle), ATGL-overexpressing (ATGL-OE) or ATGL S47A construct and treated with 50 µM etoposide for 24 h. **C**, **D** Western blot analysis of γH2AX levels was performed after 6 h of etoposide treatment. The images are representative of three independent experiments that yielded similar results. β-Actin and ATGL were used as loading and transfection controls, respectively. The data are presented as the means ± SDs from three independent experiments. Statistical significance was determined by one-way ANOVA with Tukey’s post hoc test; *p < 0.05, **p < 0.01, ***p < 0.001 vs CTRL or as indicated by brackets.

To assess the role of ATGL’s lipolytic activity in the enhanced induction of DNA damage following etoposide treatment, HepG2 cells were treated with an inhibitor of ATGL activity, ATGListatin (ATGLi), which blocks lipolytic function without altering protein structure. Specifically, inhibition of ATGL activity resulted in reduced DNA damage induction (Fig. 2A, B). The critical role of ATGL lipolytic activity in enhancing the genotoxic effect of etoposide was further supported by the overexpression of a catalytically inactive ATGL mutant (S47A), which harbors a mutation in the catalytic domain. Unlike overexpression of wild-type ATGL, overexpression of the S47A mutant did not lead to an increase in γH2AX levels, which remained comparable to those of the etoposide-treated control (Fig. 2C, D). Collectively, these results demonstrated that ATGL activity is directly associated with the sensitivity of HepG2 cells to etoposide.

### ATGL induces different post-translational regulation of p53 upon etoposide by activating the PPARα/p300 axis

**Fig. 3:**
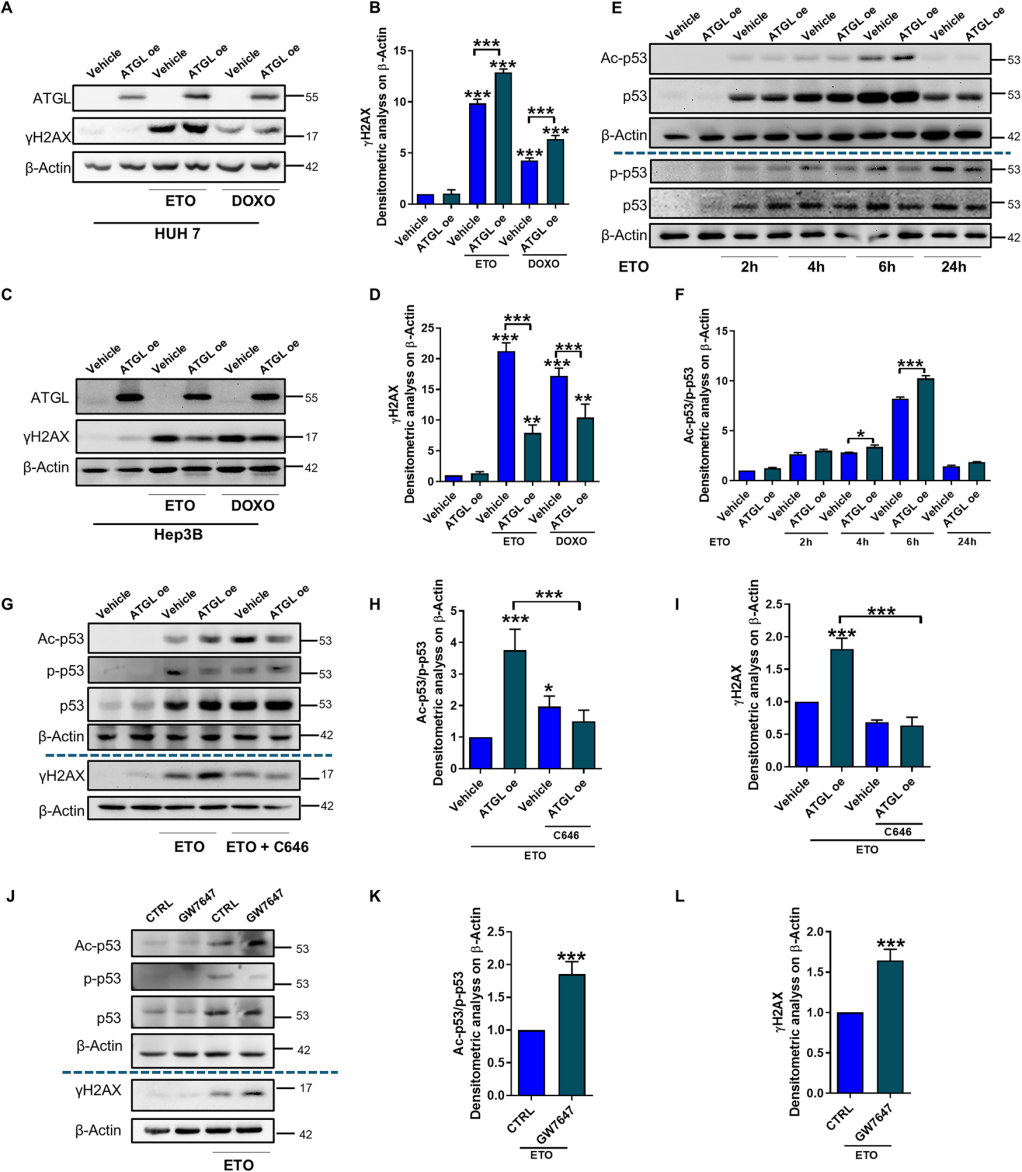
ATGL induces different post-translational regulation of p53 upon etoposide by activating the PPARα/p300 axis. **A**, **B** HUH7 and **C**, **D** Hep3B cells were transfected with an empty vector (Vehicle) or ATGL-overexpressing construct (ATGL-OE) for 24 h and then treated with 50 µM etoposide or 2 µM doxorubicin for 6 h. Western blot analysis of γH2AX levels was performed. **E** Western blot analysis of Ac-p53 Lys-382, p-p53 Ser-15 and p53 levels was performed in HepG2 cells after treatment with 50 µM etoposide for 2, 4, 6 and 24 h. **F** Densitometric ratios of Ac-p53 and p-p53 after treatment with 50 µM etoposide for 2, 4, 6 and 24 h. **G**–**I** HepG2 cells were transfected with empty vector (Vehicle) or ATGL-overexpressing (ATGL-OE) construct and, after 24 h, treated with 50 µM etoposide for 6 h with or without 10 µM C646 for 24 h. Western blot analysis of Ac-p53 Lys-382, p-p53 Ser-15 and p53 levels was performed. **H** Densitometric analysis ratios of Ac-p53 and p-p53 after treatment with 50 µM etoposide for 6 h. **J**–**L** HepG2 cells were treated with 50 µM etoposide for 6 h with or without 1 µM GW7647 for 24 h. Western blot analysis of Ac-p53 Lys-382, p-p53 Ser-15, p53 and γH2AX levels was performed. **K** Densitometric analysis of ratio between Ac-p53 and p-p53 expression after treatment with 50 µM etoposide for 6 h. The images are representative of three independent experiments that yielded similar results. β-Actin and ATGL were used as loading and transfection controls, respectively. The data are presented as the means ± SDs from three independent experiments. Statistical significance was determined by Student *t* test and one-way ANOVA with Tukey’s post hoc test; *p < 0.05, **p < 0.01, ***p < 0.001 vs CTRL or as indicated by brackets.

By evaluating the role of ATGL in the genotoxic damage response in other HCC cell lines, we observed that the effects of ATGL in HUH7 cells mirrored those observed in HepG2 cells, in both ATGL-overexpressing (Fig. 3A, B) and silenced cells (Supplementary Fig. 1A, B), whereas ATGL overexpression did not lead to a similar effect in Hep3B cells, which are p53 null (Fig. 3C, D). Thereafter, we investigated the contribution of p53, focusing on its main post-translational modifications (PTMs), which are involved in the DNA damage response. The time course experiment involving etoposide treatment revealed changes in p53 Ser-15 phosphorylation and Lys-382 acetylation after ATGL overexpression, whereas no changes in total p53 levels were observed (Fig. 3E; Supplementary Fig. 1C–E). Notably, the ratio of p53 acetylation to p53 phosphorylation significantly shifted in favor of acetylation, demonstrating a different response in p53 PTMs in the presence of high levels of ATGL (Fig. 3F). The same results were obtained in HUH7 cells (Supplementary Fig. 1F–H). First, we evaluated the activation of ATM, the main kinase contributing to p53 phosphorylation during the DNA damage response. However, no change was observed following etoposide treatment in ATGL-overexpressing cells compared with control cells (Supplementary Fig. 1I, J). Therefore, we targeted the acetyltransferase p300 known to catalyze p53 Lys-382 acetylation in response to DNA damage [4, 10]. Based on this, we exploited the selective inhibitor of p300, namely C646 (Sigma‒Aldrich, St. Louis, MO, USA) at a concentration of 10 µM 24 h before adding etoposide. The inhibition of p300 activity fully abolished ATGL-induced remodeling of p53 post-translational modifications and increased the level of γH2AX in both HepG2 (Fig. 3G–I; Supplementary Fig. 1K, L) and HUH7 cells (Supplementary Fig. 1M, N), confirming the critical role of p53 acetylation in the response to DNA damage in the context of ATGL overexpression.

The lipolytic activity of ATGL is responsible for the release of FAs, which can activate downstream transcription factors, including members of the PPAR family [11]. In particular, PPARα is known to activate p300 expression, and we observed that the use of a PPARα agonist (GW7647) upon etoposide treatment recapitulates the phenotype observed upon ATGL overexpression, both in terms of p53 PTMs and γH2AX levels in both HepG2 (Fig. 3J–L; Supplementary Fig. 2A) and HUH7 cells (Supplementary Fig. 2B, C). These findings suggest that ATGL modulates p53 acetylation and the DNA damage response through activation of the PPARα/p300 axis.

### ATGL levels are predictive of the commitment of p53 to apoptosis genes upon etoposide

**Fig. 4:**
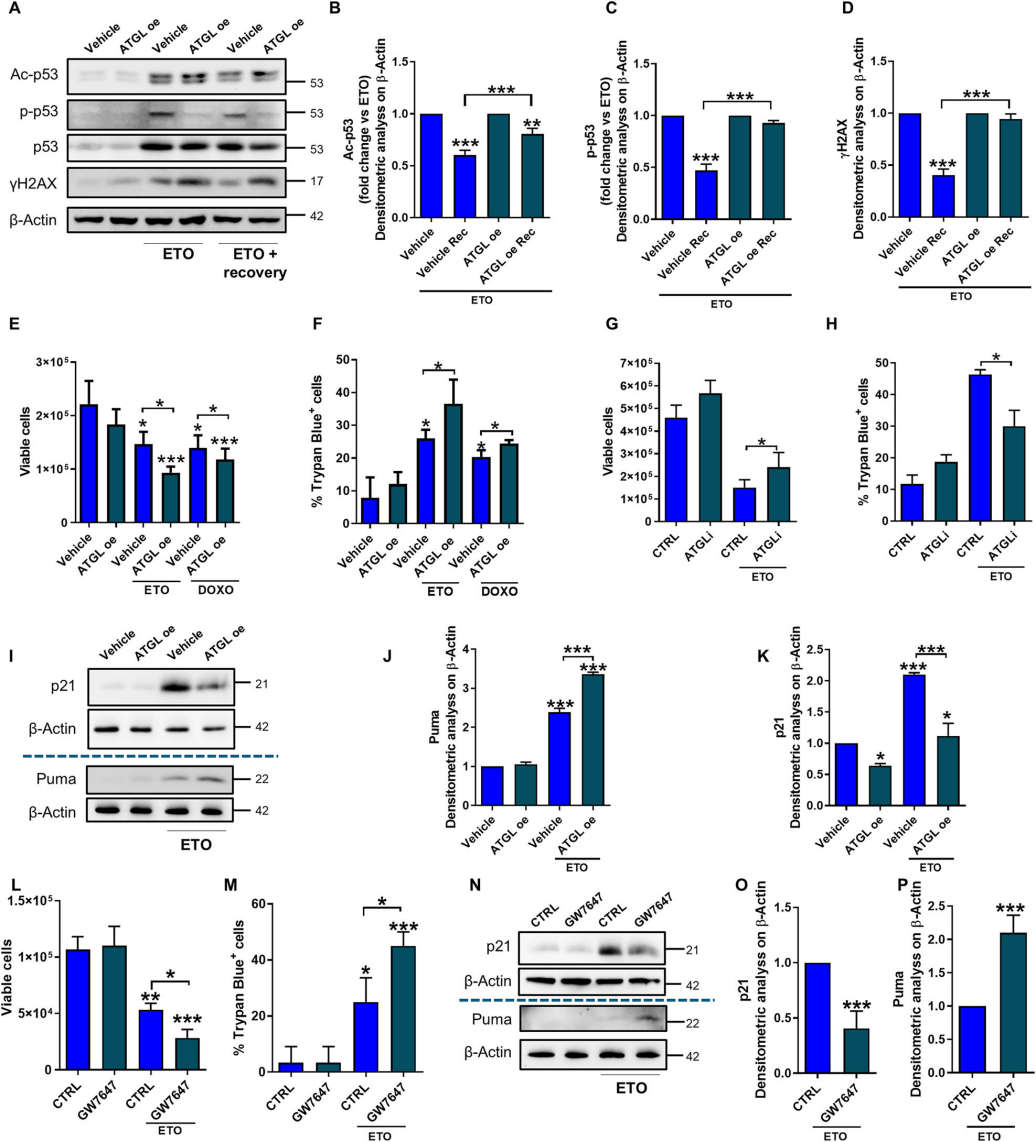
ATGL levels are predictive of the commitment of p53 to apoptosis genes upon etoposide. **A**–**D** HepG2 cells were transfected with empty vector (Vehicle) or ATGL-overexpressing construct (ATGL-OE) and, after 24 h, treated with 50 µM etoposide for 6 h with or without recovery (Rec) with fresh medium for 2 h. Western blot analysis of Ac-p53 Lys-382, p-p53 Ser-15, p53 and γH2AX levels was performed. **E**, **F** HepG2 cells were transfected with empty vector (Vehicle) or ATGL-overexpressing construct (ATGL-OE) and, after 24 h, treated with 50 µM etoposide or 2 µM doxorubicin for 6 h. The proliferation was assayed via the Trypan blue direct counting procedure. **G**, **H** HepG2 cells were treated with 50 µM etoposide for 6 h with or without 25 µM ATGListatin (ATGLi) for 24 h. HepG2 cells were treated with 50 µM etoposide for 6 h. **I**–**K** Western blot analysis of p21 and Puma levels was performed. HepG2 cells were treated with 1 µM GW7647 for 24 h. **L**, **M** Proliferation was assayed by the Trypan blue direct counting procedure. **N**–**P** Western blot analysis of p21 and Puma levels was performed. The images are representative of three independent experiments that yielded similar results. β-Actin and ATGL were used as loading and transfection controls, respectively. The data are presented as the means ± SDs from three independent experiments. Statistical significance was determined by one-way ANOVA with Tukey’s post hoc test; *p < 0.05, **p < 0.01, ***p < 0.001 vs CTRL or as indicated by brackets.

To determine whether the increased levels of γH2AX after treatment of ATGL-overexpressing cells are due to altered DNA repair mechanisms, we assessed the recovery capacity for DNA damage after 2 h of etoposide treatment. While control cells presented reduced γH2AX levels after drug withdrawal, ATGL-overexpressing cells maintained the same extent of γH2AX. Similarly, p53 PTMs persisted in ATGL-overexpressing cells after recovery, whereas both p53 phosphorylation and acetylation were significantly reduced in control cells (Fig. 4A–D).

To investigate whether the impaired DNA damage recovery observed in ATGL-overexpressing cells translated into increased cell death upon etoposide exposure, we assessed viability after treating cells for 24 h with genotoxic drugs. We observed that ATGL-overexpressing cells exhibited increased sensitivity to both etoposide and doxorubicin (Fig. 4E, F; Supplementary Fig. 2D). The use of ATGLi (Fig. 4G, H) and the overexpression of a catalytically inactive ATGL mutant (Supplementary Fig. 2E–G) enabled us to demonstrate that this effect was dependent on ATGL lipase activity. Moreover, despite ATGL overexpression, the inhibition of ATGL activity prevented the cytotoxic outcome (Supplementary Fig. 2H).

The results obtained thus far suggest that the state of p53 PTMs induced by ATGL overexpression induces a preferential transcriptional response toward cell death pathways. Thus, we analyzed key p53 target genes involved in the DNA damage response: p21, which promotes cell cycle arrest to allow DNA repair together with GADD45 [12, 13], and Puma, which promotes apoptosis [14]. We found that ATGL overexpression increased Puma expression, whereas p21 levels (Fig. 4I–K) and GADD45a nuclear foci (Supplementary Fig. 3A) were decreased upon etoposide treatment.

In parallel, the finding that the PPARα agonist has the same effect as ATGL on etoposide sensitivity (Fig. 4L, M), including Puma upregulation and p21 downregulation (Fig. 4N–P), indicates that cells with increased ATGL/PPARα pathway activity are more prone to undergo apoptosis rather than cell cycle arrest for DNA repair upon etoposide treatment.

### ATGL-associated transcriptional programs in HCC human specimens are linked to PPARα/p300 signaling

**Fig. 5:**
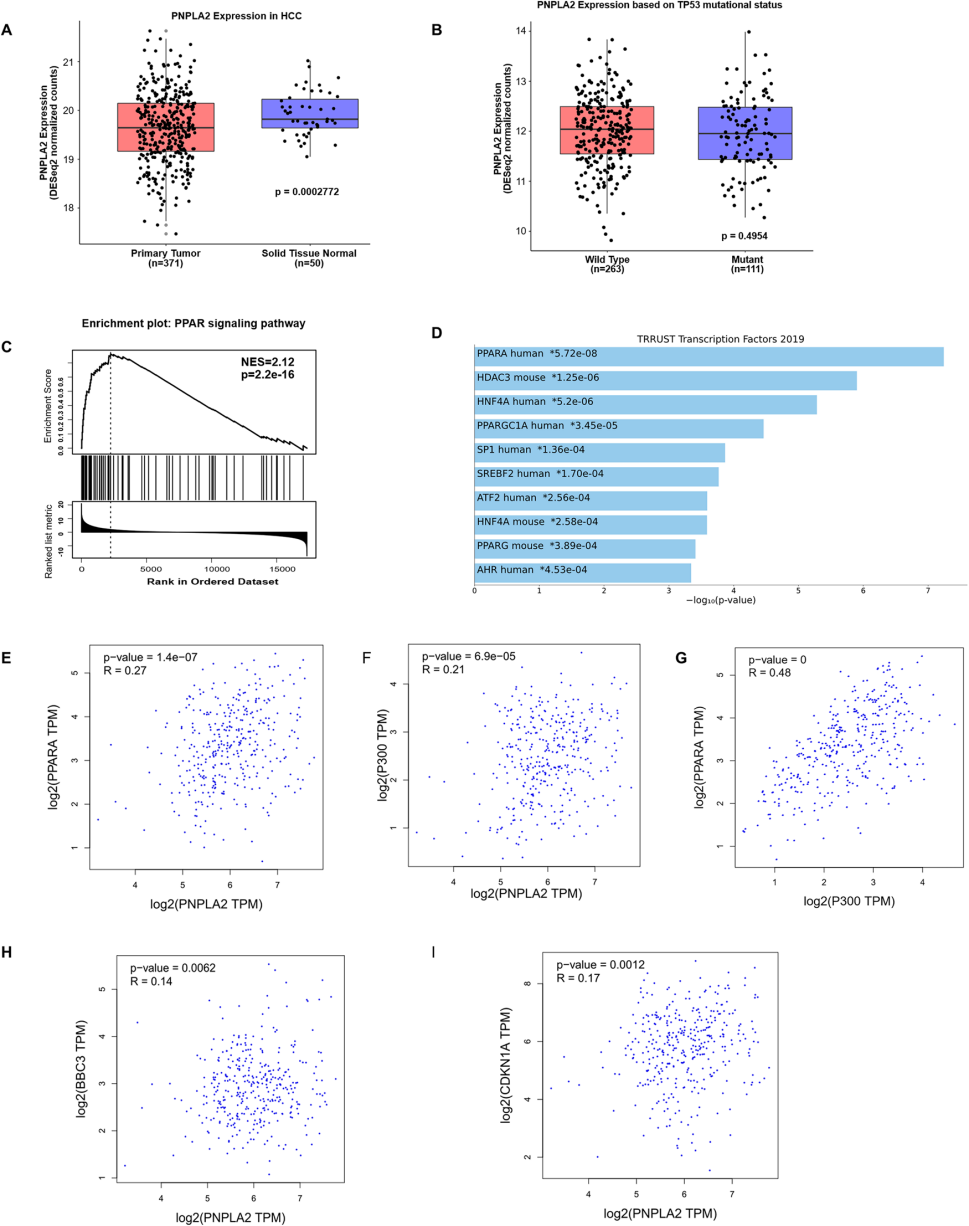
ATGL-associated transcriptional programs in HCC are linked to PPARα/p300 signaling and apoptotic commitment. **A** Boxplot showing significantly reduced *PNPLA2* expression in primary HCC tissues (primary tumor; *n* = 371) compared with solid tumor-adjacent non-tumoral liver tissues (solid tissue normal; *n* = 50) samples based on TCGA data. **B** Boxplot of Z-score–normalized ATGL expression from TCGA-LIHC RNA-seq data based on *TP53* mutation status (wild type *n* = 263 and mutant *n* = 111). **C** Visualization of the PPAR signaling pathway, reporting normalized enrichment score (NES) and adjusted *p*-value. **D** Bar plot showing the most significantly enriched transcription factors after a transcription factor enrichment analysis performed using TRRUST transcription factors 2019 database on differentially expressed genes (DEGs) in ATGL-high versus ATGL-low HCC samples. Adjusted *p*-value was reported. Scatter plot showing the correlation between **E** *PNPLA2* and PPARα (*PPARA*); between **F** *PNPLA2* and *EP300*; between **G** *PPARA* and *EP300*; between **H** *PNPLA2* and Puma (*BBC3*); between **I** *PNPLA2* and p21 (*CDKN1A*) mRNA expression levels in HCC samples from the TCGA-LIHC cohort analyzed using GEPIA. Gene expression values are reported as log2-transformed TPM. Each dot represents an individual tumor sample.

To validate the mechanistic evidence obtained in vitro, we interrogated transcriptomic data derived from human HCC biopsies (TCGA-LIHC dataset) revealing that *PNPLA2* (ATGL) is significantly reduced in tumoral tissues compared with normal liver samples (Fig. 5A). Consistently, Gene Set Enrichment Analysis (GSEA) revealed a significant enrichment of the Gene Ontology (GO) terms related to the positive regulation of the cell cycle in HCC samples with low PNPLA2 expression (Supplementary Fig. 3B). No significant differences in ATGL expression were detected between *TP53* wild-type and mutant tumors (Fig. 5B), indicating that ATGL transcriptional levels are independent of p53 mutational status in HCC.

By performing GO analysis on the top 50 genes positively correlated with PNPLA2 expression (Supplementary Fig. 3C), apart from metabolic signatures including lipid metabolism and mitochondrial function, we highlighted the presence of the term PPAR signaling, that is also particularly enriched in samples with high ATGL levels by GSEA (Fig. 5C; Supplementary Fig. 3D). Notably, PPARα was the most significantly enriched transcription factor involved in the regulation of genes associated with ATGL (Fig. 5D). The reciprocal and positive correlation between *PNPLA2* and *PPARA* and *EP300* in biopsies further supports the relevance of ATGL-PPARα–p300 signaling axis in human HCC (Fig. 5E–G). Finally, in agreement with in vitro experiments the GSEA identified that tumors characterized by low ATGL expression were preferentially enriched in genes involved in different types of genotoxic stresses (Supplementary Fig. 3E), a significant positive correlation was demonstrated between ATGL and the p53 target genes *BBC3* (PUMA) (Fig. 5H) and *CDKN1A* (p21) (Fig. 5I).

## Discussion

Given our previous characterization of the effects of increased oxidative metabolism driven by ATGL overexpression in HCC cells, we elucidated the molecular mechanism by which ATGL may increase DNA damage in response to genotoxic agents. We observed that ATGL expression levels are predictive of the response to DNA damage, as assessed by H2AX phosphorylation, in both the HepG2 and HUH7 cell lines. Etoposide-induced DNA damage increases progressively up to 6 h of treatment, with ATGL-overexpressing cells exhibiting increased levels of damage. By 24 h, the levels of DNA damage markers, including γH2AX and p53, began to decrease, which correlated with the onset of downstream effects, namely, cell death. Accordingly, a recent study also demonstrated that vimentin modulates ATGL-mediated lipolysis, thereby limiting the severity of damage [15].

The relationship between the DNA damage response and lipid metabolism has been explored across various cellular systems [16, 17]. DNA damage has been shown to induce a rearrangement of lipid metabolism, thereby promoting inflammation [17]. Conversely, the lipid composition of cancer cells can influence genomic instability by facilitating DNA damage or modulating its repair. Notably, the number of intracellular lipid droplets has emerged as a prognostic factor for the response to genotoxic therapies in various systems, including multiple myeloma [18] and prostate cancer [19]. Furthermore, increased fatty acid oxidation has been associated with increased DNA damage, improved chemotherapeutic response, and reversal of obesity-associated chemoresistance [20]. Increased lipolytic activity impacts lipid homeostasis in distinct ways: by reducing the size of lipid droplets, thus preventing the sequestration of lipophilic chemotherapeutic agents [21]; by promoting the oxidation of FAs; and by activating downstream transcription factors via released FAs.

Interestingly, ATGL-dependent damage in HCC was opposite to that in p53-null Hep3B hepatocellular carcinoma cells, a relevant result that needs further investigation and suggests both p53-dependent and p53-independent mechanisms.

P53 is known to be involved not only in the repair response, which promotes cell cycle arrest through the induction of genes such as p21 and GADD45A but also in the proapoptotic response, which is triggered by the expression of genes such as Puma [22–24]. The expression of one set of genes over another directs the cell either toward DNA damage repair, when damage is limited, or toward cell death, if the extent of damage exceeds the cell’s repair capacity. In this context, phosphorylation at serine 15, a key event in the DNA damage response, is also capable of manipulating p53 transcriptional activity [25, 26]. Indeed, this PTM primarily promotes cell cycle arrest by reducing p53 nuclear export and consequently increasing its nuclear localization [26, 27]. Our previous report linked ATGL activity to altered p53 acetylation levels [4]. In HepG2 and HUH7 ATGL-overexpressing cells treated with etoposide, the ratio of p53 acetylation to phosphorylation was skewed toward acetylation. These findings suggest that ATGL influences the post-translational modification landscape of p53 by favoring acetylation over phosphorylation. This result is in agreement with the increased lysine acetylation of proteins in various cellular models, as FAs released via ATGL provide substrates for acetyl-CoA generation [28, 29].

On the other hand, polyunsaturated FAs with 18 to 22 carbon atoms that are produced by ATGL activate PPARα [7, 8, 30], which is a positive regulator of the acetyltransferase p300. Importantly, the relevance of this signaling axis is supported by patient-derived transcriptomic data that revealed a significant positive association between *PNPLA2*, *PPARA* and *EP300* expression. Here, we demonstrated that p300 is required for both the increased DNA damage and the enhanced sensitivity to etoposide observed in ATGL-overexpressing cells. This finding is consistent with the recapitulation of the ATGL-induced phenotype upon treatment of HCC cells with a PPARα agonist, which alone increased DNA damage in response to etoposide and reprogrammed p53 post-translational modifications.

Under our experimental conditions, ATGL/PPARα-induced reprogramming of p53 PTMs resulted in increased levels of cell death in ATGL-overexpressing cells, as confirmed by increased expression of the proapoptotic protein Puma. Moreover, the expression of p21, a protein that mediates cell cycle arrest in response to DNA damage to facilitate repair [31], was reduced. Notably, the pharmacological activation of PPARα mimics the impact of ATGL overexpression on the same p53 downstream targets. Finally, the recovery experiment revealed a delay in DNA damage repair due to reduced p21 expression and GADD45 nuclear accumulation. This represents a critical point, as it promotes cell death. Consistently, the enrichment of pathways related to genotoxic stress in PNPLA2-low tumors suggests that HCC samples with reduced ATGL expression are transcriptionally primed to activate repair mechanisms in response to DNA damage. In contrast, higher ATGL levels are associated with attenuation of these pathways, supporting our experimental model in which ATGL overexpression enhances DNA damage and apoptotic signaling upon genotoxic stress.

Overall, our data indicates that the stimulation of ATGL activity and PPARα signaling can improve those therapeutic strategies based on DNA damaging agents that are known to be particularly unsuccessful in the treatment of HCC patients [32]. Although in vivo validation will be necessary to assess the impact of ATGL-dependent modulation in the DNA damage response, the human transcriptomic analyses support the importance of the ATGL/PPARα/p300 signaling axis in HCC.

## Conclusions

This study highlights that the enzymatic activity of ATGL and the downstream activation of the PPARα/p300 axis play a role in the genotoxic stress response in HCC. These findings support the tumor-suppressive role of ATGL in HCC, not only through its previously described metabolic functions but also by influencing the cellular response to genotoxic agents. Assessing ATGL levels may be important for patient stratification, enabling more accurate prediction of therapeutic strategies dependent on p53 expression in HCC. Moreover, enhancing ATGL activity or using PPARα agonists may represent a promising approach to improve the efficacy of current treatments.

## Materials and methods

### Materials

Albumin (A3782), ATGListatin (5.30151), C646 (382113), crystal violet (61135), doxorubicin (DOXO) (D5220), EDTA (E6758), EGTA (E4378), etoposide (ETO) (341205), GW7647 (370698), KH_2_PO_4_ (A0261677), MgCl_2_ (A748033 012), Formalin solution neutral buffered 10% (HT5012), sodium deoxycholate (D6750), sodium orthovanadate (S6508), sodium pyrophosphate tetrabasic decahydrate (30411), sucrose (S0389), and Triton X-100 (T9284) were obtained from Sigma‒Aldrich, St. Louis, MO, USA. Trypan blue 0.4% solution (17-942E) was obtained from Lonza, Antwerp, Belgium. Goat anti-mouse (172-1011) and anti-rabbit (172-1019) horseradish peroxidase (H + L)-conjugated IgG were obtained from Bio-Rad Laboratories, Hercules, California. Hoechst 33342 (H1399) was purchased from Thermo Fisher Scientific. DTT (281) and protease inhibitor cocktail were obtained from AMRESCO, Radnor, Pennsylvania. Tris-base (1610716) and sodium dodecyl sulfate (SDS) (161-0300) were obtained from Bio-Rad. TEMED (110189) was obtained from PanReac AppliChem, Barcelona, Spain.

### Cell lines and treatments

HepG2, HUH7 and Hep3B cell lines were purchased from American Type Culture Collection (ATCC). HepG2 and HUH7 cells were grown in RPMI 1640 (EuroClone, Milan, Italy), and Hep3B cells were grown in Dulbecco’s modified Eagle’s medium (DMEM) supplemented with 1 g/L glucose (EuroClone). All media were supplemented with 10% fetal bovine serum (FBS) (EuroClone), 10 U/mL penicillin/streptomycin (EuroClone) and 2 mM L-glutamine (EuroClone). The cells were authenticated and characterized by the supplier. Mycoplasma tests were routinely carried out according to protocols from our laboratory. The cells were cultured at 37 °C in an atmosphere of 5% CO_2_ in the air and plated at a density of 2 × 10^5^ cells/mL for all the experiments. After 24 h of incubation, the cells were treated with etoposide (Sigma‒Aldrich, cat. number 341205) and doxorubicin (Sigma‒Aldrich, cat. number D5220) for the indicated hours.

The cells were treated with 25 μM ATGListatin (Sigma‒Aldrich, cat number 5.30151) for 24 h, 10 μM C646 (Sigma‒Aldrich, cat. number 382113) for 24 h, and 1 μM GW7647 (Sigma‒Aldrich, cat. number 370698) for 24 h.

Twenty-four h after plating, the cells were transiently transfected with pcDNA™4/HisMaxC, pcDNA™4/HisMaxC-ATGL or pcDNA™4/HisMaxC-ATGL (S47A) for 48 h with polyethylenimine (PEI) reagent according to the manufacturer’s instructions. The pcDNA™4/HisMaxC and pcDNA™4/HisMaxC-ATGL plasmids were kindly provided by Prof. Rudolf Zechner, Institute of Molecular Biosciences, Karl-Franzens-Universität Graz, Graz (Austria); the pcDNA™4/HisMaxC-ATGL (Ser47Ala) plasmid was obtained as previously described [33]. The transfection efficiency was determined by transfecting cells with pATGL-EGFP. The percentage of transfected cells was assessed as >85%. siATGL was reversely transfected using Lipofectamine® RNAiMAX Transfection Reagent (Thermo Fisher Scientific, cat. number 13778150) according to the manufacturer’s protocol.

### Western blot analysis

At the end of the experiment, the cells were resuspended in lysis buffer (50 mM Tris-HCl, pH 7.4; 150 mM NaCl; 1 mM EDTA; 1% Triton X-100; 0.5% sodium deoxycholate; 0.1% SDS; 10 mM NaF; 5 mM sodium pyrophosphate; 2 mM sodium orthovanadate) supplemented with a protease inhibitor cocktail (AMRESCO). After centrifugation at 14,000×*g* for 15 min, the Lowry et al. [34] method was used to determine the protein concentration before electrophoresis by SDS‒PAGE and blotting onto a nitrocellulose membrane (Bio‒Rad).

The following primary antibodies were used: β-Actin (Cell Signaling Technology, cat. number #4970S, diluted 1:1000), γH2AX Ser-139 (Cell Signaling Technology, cat. number #9718, diluted 1:1000), ATGL (Cell Signaling Technology, cat. number 2138S, diluted 1:1000), pATM Ser-1981 (Cell Signaling Technology, cat. number #5883, diluted 1:1000), ATM (Cell Signaling Technology, cat. number #2873, diluted 1:1000), p21 (Cell Signaling Technology, cat. number #2947, diluted 1:1000), PPARα (Santa Cruz Biotechnology, cat. number sc-398394, diluted 1:1000), Puma (Cell Signaling Technology, cat. number #4976, diluted 1:1000), Ac-p53 Lys-382 (Cell Signaling Technology, cat. number #2525S, diluted 1:1000), p-p53 Ser-15 (Cell Signaling Technology, cat. number #9284S, diluted 1:1000), and p53 (Sigma-Aldrich, cat. number #P5813, diluted 1:1000). After incubation with specific horseradish peroxidase (HRP)-conjugated secondary antibodies (Bio-Rad), ImageJ software was used to perform densitometry analyses.

### Cell proliferation assays

Cell proliferation was evaluated via the Trypan blue exclusion test procedure and a Crystal violet colorimetric assay (Sigma‒Aldrich cat. number 61135). For the crystal violet assay, the cells were fixed with a formalin solution containing 10% neutral buffer (containing 4% paraformaldehyde) for 10 min, washed with PBS and stained with 0.1% crystal violet for 30 min at RT. After three washes with PBS, the cells were air-dried. The elution of Crystal Violet was performed with 10% methanol, and the quantification was performed by measuring the absorbance at 595 nm via a microplate reader.

### Fluorescence microscopy analysis

The medium was removed, and the cells were fixed with a neutral buffered 10% formalin solution for 10 min. After washing with PBS, they were permeabilized with PBS/0.1% Triton X-100 solution for 10 min. The cells were blocked with PBS/10% FBS solution for 1 h and incubated overnight with anti-γH2AX Ser-139 (Cell Signaling Technology, cat. number #9718) or GADD45A (Cell Signaling Technology, cat. number #4632) antibodies and then incubated for 1 h with an Alexa Fluor™ 594 donkey anti-rabbit IgG (H + L) secondary antibody; the nuclei were stained with 1 µg/mL Hoechst 33342 for 10 min. A Delta Vision Restoration Microscopy System (Applied Precision, Issaquah, WA) equipped with an Olympus IX70 fluorescence microscope (Olympus Italia, Segrate, Milano, Italy) was used to acquire fluorescence images of the cells. The fluorescence intensity was evaluated via ImageJ software.

### Bioinformatic analyses

RNA-seq data of HCC and normal liver tissues were obtained from The Cancer Genome Atlas (TCGA) (TCGA-LIHC dataset). Raw RNA-seq counts of primary tumors and adjacent normal tissues were normalized using DESeq2. TP53 mutational status of TCGA-LIHC samples was obtained from cBioPortal. Statistical significance between groups was assessed using a two-tailed Wilcoxon rank-sum test. Data are presented as boxplots with individual samples shown as jittered points, and *p*-values ≤ 0.05 were considered statistically significant.

Gene expression correlation analysis was performed on TCGA-LIHC RNA-sequencing data using Pearson correlation coefficients to identify genes positively associated with PNPLA2 (ATGL) gene expression (LinkedOmics) [35]. The top 50 positively correlated genes were subjected to Gene Ontology over-representation analysis to identify enriched biological pathways (Webgestalt) [36]. Statistical significance was evaluated as previously described [35], and enriched terms with *p*-value < 0.05 were considered significant. Multiple-test correction is performed using the Benjamini and Hochberg method to generate the False Discovery Rate (FDR). LinkInterpreter was used to evaluate functional enrichment (WikiPathways database). Gene expression correlation analysis was performed using the GEPIA web server [37], which integrates RNA-sequencing data from TCGA. PNPLA2 (ATGL) and PPARA, BBC3 and P300 mRNA expression levels were extracted from the TCGA-LIHC dataset. Expression data were normalized as transcripts per million (TPM) and log2-transformed prior to analysis. Correlation between gene expression levels was assessed using correlation statistics implemented in GEPIA, and statistical significance was determined using two-tailed tests. Correlation coefficients (*R*) (Pearson test) and corresponding *p*-values are reported. Transcription factor enrichment analysis of genes over-expressed in ATGL-high HCC was performed using the Enrichr platform [38–40]. TRRUST Transcription Factors 2019 was used to identify enriched transcription factor–target gene relationships, including PPARα, while TF–PPI analysis was applied to identify associated transcriptional co-regulators. Statistical significance was evaluated by Fisher’s exact test with Benjamini–Hochberg correction.

Gene set enrichment analysis (GSEA) was performed using TCGA-LIHC RNA-seq data. Samples were ranked according to *PNPLA2* expression and divided into two groups: *PNPLA2* high, containing the top 25% of the ranking, and *PNPLA2* low, containing the bottom 25% of the ranking. GSEA was performed using GSEA v4.4.0 software (https://www.gsea-msigdb.org) [41] to analyze enriched pathways in the two groups.

### Data analysis

The results are expressed as the means ± SDs of data derived from at least 3 independent experiments. Student’s *t* test for comparisons was used for statistical analyses of only two variables, and one-way ANOVA with post hoc Tukey tests was used for multiple comparisons. GraphPad Prism 8 software was used to create graphics and perform the statistical analysis. Comparisons were considered statistically significant at *p* ≤ 0.05 (*), very statistically significant at *p* ≤ 0.01 (**) and extremely statistically significant at *p* ≤ 0.001 (***).

## Supplementary information

Supplementary Materials

## Data Availability

Not applicable
