# Supplementary material for: ATGL sensitizes hepatocellular carcinoma cells to genotoxic drugs by modulating p53 acetylation/phosphorylation status
Source: Cell Death Discov. 2026 Mar 20;12:164. doi: 10.1038/s41420-026-03048-4 (PMC13040076; doi:10.1038/s41420-026-03048-4)
Supplement: Supplementary file 1 — Supplementary Materials [file 41420_2026_3048_MOESM1_ESM.docx]

**Supplementary Materials**

**ATGL sensitizes hepatocellular carcinoma cells to genotoxic drugs by modulating p53 acetylation/phosphorylation status.**

**Serena Castelli^1,2^, Angela De Cristofaro^3^, Enrico Desideri^4^, Emanuele Salvi^3^, Fabio Ciccarone^2,3^**^#^ **and Maria Rosa Ciriolo^2,3,^** ^#,*^

^1^ Department for the Promotion of Human Science and Quality of Life, San Raffaele Open University, Via di Val Cannuta, 247, 00166 Rome, Italy

^2^ IRCCS San Raffaele Roma, 00166 Rome, Italy

^3^ Department of Biology, University of Rome Tor Vergata, 00133 Rome, Italy. ADC and ES are enrolled in PhD Program in Cellular and Molecular Biology, Department of Biology, University of Rome Tor Vergata, Rome, Italy.

^4^ Department of Life Sciences, Health and Health Professions, Link Campus University, Via del Casale di San Pio V, 44, 00165 Rome, Italy

^#^MRC and FC equally contributed as co-last authors.

*Corresponding author; ciriolo@bio.uniroma2.it


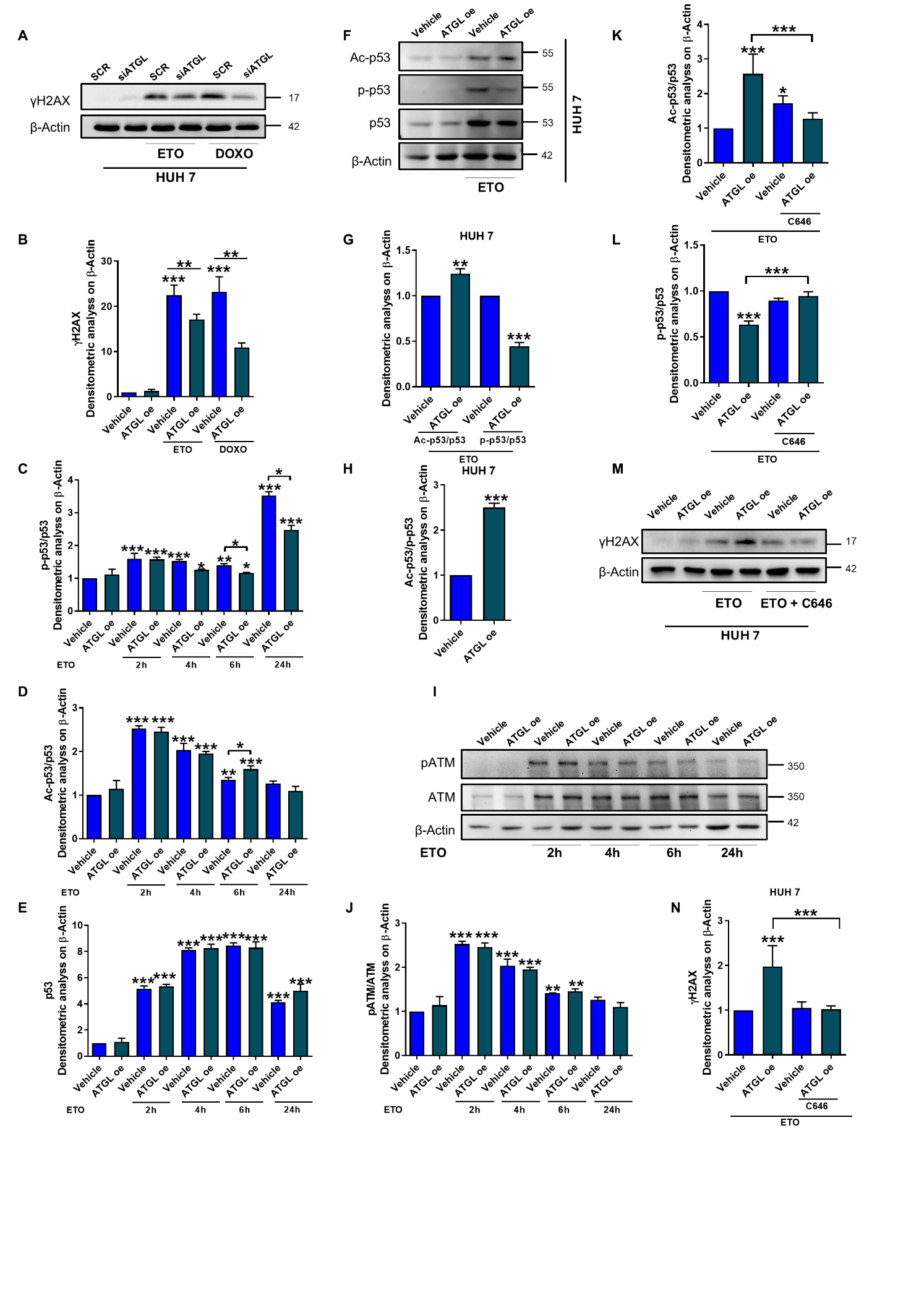


**Supplementary Figure 1.** HUH7 cells were silenced for ATGL (siATGL; SCR=Scramble) and, after 24 h, treated with 50 µM etoposide or 2 µM doxorubicin for 6 h. (**A**, **B**) Western blot analysis of γH2AX levels was performed. (**C-E**) Densitometric analysis of Ac-p53 Lys-382, p-p53 Ser-15 and p53 shown in Figure 3E. (**F, G**) HUH7 cells were transfected with empty vector (Vehicle) or ATGL-overexpressing construct (ATGL-OE) and after 24h treated with 50 µM etoposide for 6h. Western blot analysis of Ac-p53 Lys-382, p-p53 Ser-15 and p53 levels was performed. (**H**) Ratio between the densitometric analyses of Ac-p53 and p-p53 after treated with 50 µM etoposide for 6h. (**I**, **J**) Western blot analysis of pATM Ser-1981 and ATM levels was performed on HepG2 cells after treated with 50 µM etoposide for 2, 4, 6 and 24h. (**K**, **L**) Densitometric analysis of Ac-p53 Lys-382, p-p53 Ser-15 and p53 shown in Figure 3G. (**M, N**) HUH7 cells were transfected with empty vector (Vehicle) or ATGL-overexpressing (ATGL-OE) construct and, after 24 h, treated with 50 µM etoposide for 6 h with or without 10 µM C646 for 24 h. Western blot analysis of γH2AX levels was performed.


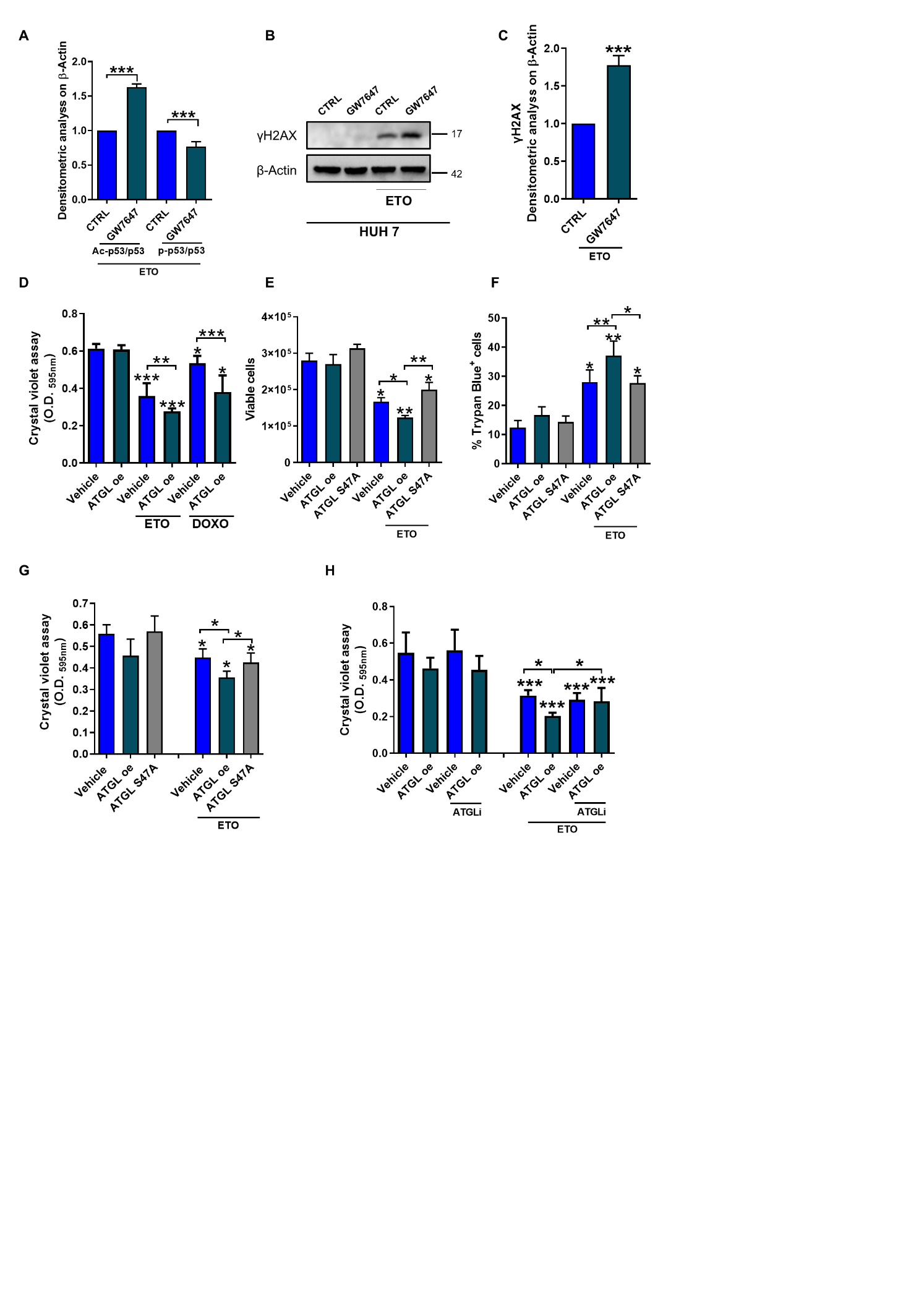


**Supplementary Figure 2.** (**A**) Densitometric analysis of Ac-p53 Lys-382, p-p53 Ser-15 and p53 shown in Figure 3J. (**B, C**) HUH7 cells were treated with 50 µM etoposide for 6 h with or without 1 µM GW7647 for 24 h. Western blot analysis of γH2AX levels was performed. β-Actin was used as loading control. Data are presented as mean ± SD from three independent experiments. Statistical significance was determined by one-way ANOVA with Tukey’s post-hoc test; *p < 0.05, **p < 0.01, ***p < 0.001. HepG2 cells were transfected with empty vector (Vehicle), ATGL-overexpressing construct (ATGL-OE) construct and after 24h treated with 50 µM etoposide (ETO) or 2 µM doxorubicin (DOXO) for 24h. (**D**) The proliferation was assayed by Crystal violet assay. HepG2 cells were transfected with empty vector (Vehicle), ATGL-overexpressing (ATGL-OE) or ATGL S47A and after 24h treated with 50 µM etoposide for 24h. The proliferation was assayed by (**E, F**) Trypan Blue direct counting procedure and (**G**) Crystal violet assay. HepG2 cells were transfected with empty vector (Vehicle) or ATGL-overexpressing construct (ATGL-OE) and after 24h were treated with 50 µM etoposide for 6h with or without 25 µM ATGListatin (ATGLi) for 24h. (**H**) The proliferation was assayed by Crystal violet assay.


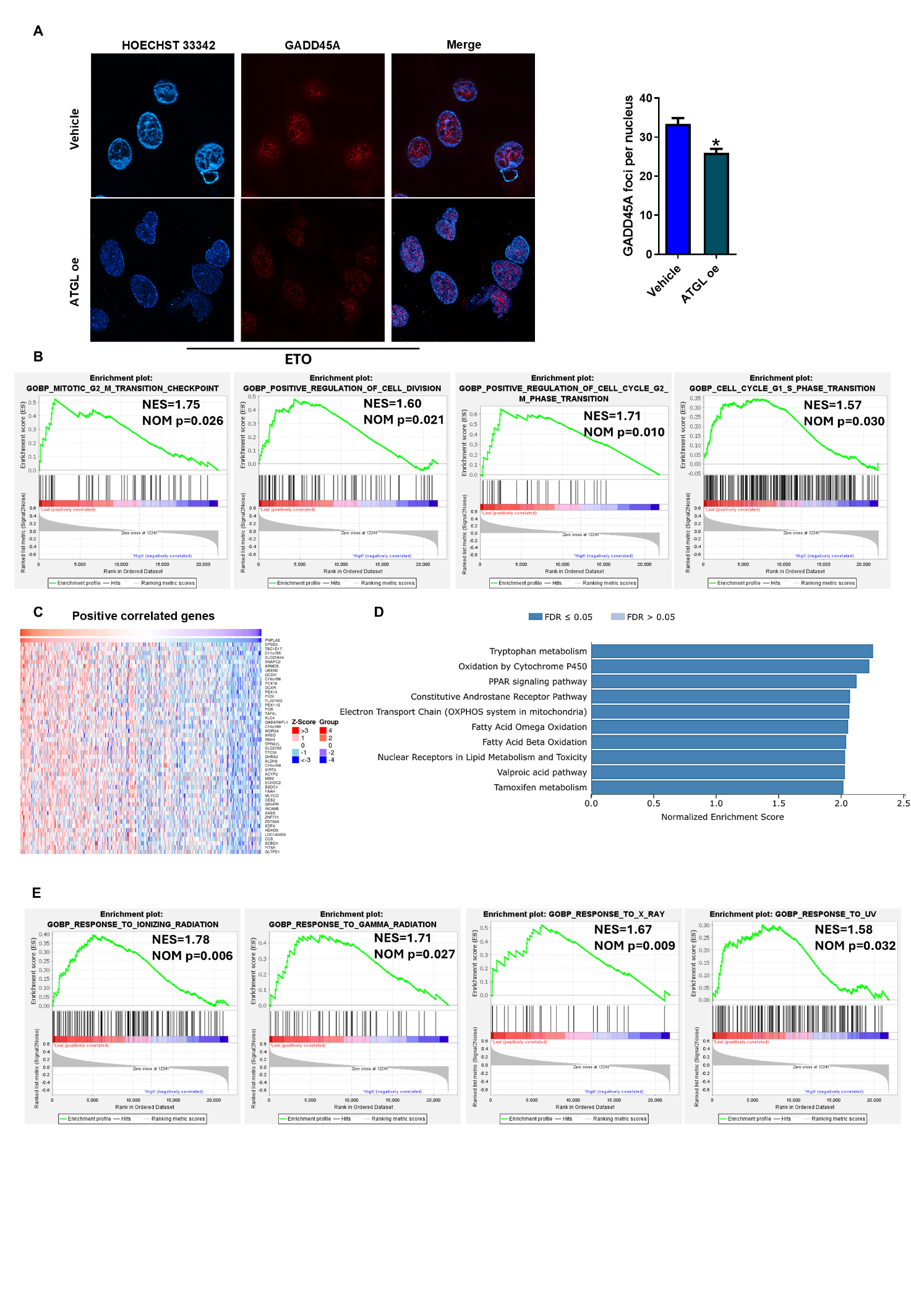


**Supplementary Figure 3.** (**A**) Representative immunofluorescence images and quantification of GADD45A-foci per nucleus in HepG2 cells treated with 6h etoposide. Data are presented as mean ± SD from three independent experiments. Statistical significance was determined by one-way ANOVA with Tukey’s post-hoc test; *p < 0.05, **p < 0.01, ***p < 0.001. (**B, E**) Gene Set Enrichment Analysis (GSEA) comparing PNPLA2-low versus PNPLA2-high tumours of the TCGA-LIHC dataset using gene sets related to cell cycle regulation, including G2/M transition, cell division, G1/S transition, and G2/M phase transition (**B**), as well as pathways involved in the cellular response to genotoxic stress,such as response to ionizing radiation, gamma radiation, X-ray, and UV (**E**). Normalized enrichment score (NES) and nominal p-values (NOM p) are reported for each gene set.

(**C**) Heatmap showing the top 50 genes positively correlated with PNPLA2 in LIHC from LinkedOmics. (**D**) Gene Ontology (GO) enrichment analysis for Biological Processes performed using LinkInterpreter on the TCGA-LIHC dataset, showing the most significantly enriched pathways ranked by false discovery rate (FDR). Gene Set Enrichment Analysis (GSEA) performed on TCGA-LIHC transcriptomic data comparing ATGL-low versus ATGL-high tumors using Gene Ontology Biological Process (GO-BP) gene sets.
